# The Southern Ocean in the Coupled Model Intercomparison Project phase 5

**DOI:** 10.1098/rsta.2013.0296

**Published:** 2014-07-13

**Authors:** A. J. S. Meijers

**Affiliations:** British Antarctic Survey, High Cross, Madingley Road, Cambridge CB3 0ET, UK

**Keywords:** Southern Ocean, CMIP5, Antarctic circumpolar current, meridional overturning circulation, mixed layer, sea ice

## Abstract

The Southern Ocean is an important part of the global climate system, but its complex coupled nature makes both its present state and its response to projected future climate forcing difficult to model. Clear trends in wind, sea-ice extent and ocean properties emerged from multi-model intercomparison in the Coupled Model Intercomparison Project phase 3 (CMIP3). Here, we review recent analyses of the historical and projected wind, sea ice, circulation and bulk properties of the Southern Ocean in the updated Coupled Model Intercomparison Project phase 5 (CMIP5) ensemble. Improvements to the models include higher resolutions, more complex and better-tuned parametrizations of ocean mixing, and improved biogeochemical cycles and atmospheric chemistry. CMIP5 largely reproduces the findings of CMIP3, but with smaller inter-model spreads and biases. By the end of the twenty-first century, mid-latitude wind stresses increase and shift polewards. All water masses warm, and intermediate waters freshen, while bottom waters increase in salinity. Surface mixed layers shallow, warm and freshen, whereas sea ice decreases. The upper overturning circulation intensifies, whereas bottom water formation is reduced. Significant disagreement exists between models for the response of the Antarctic Circumpolar Current strength, for reasons that are as yet unclear.

## 1. Introduction

The Southern Ocean is the site of a climatically important interface between the ocean interior, the atmosphere and the cryosphere. In this vast region, the combination of strong surface fluxes of heat and freshwater, along with powerful and persistent westerly winds, act to tilt isopycnals meridionally. This density gradient drives the Antarctic Circumpolar Current (ACC) and provides a low-resistance pathway from the deep ocean to the surface that allows the upwelling, surface transformation and subsequent subduction of the large volumes of water that form the southern limb of the global Meridional Overturning Circulation (MOC).

The MOC itself acts to transport heat and freshwater into the ocean interior, as well as supporting the exchange of gases such as oxygen and carbon dioxide between the deep ocean interior and the atmosphere. The existence of these pathways and the strong ocean–atmosphere coupling means that the Southern Ocean makes a disproportionately large contribution to maintaining the global climate [1,2].

The ACC and MOC are complex emergent features of the ocean circulation controlled by the balance between wind momentum input, the buoyancy gradient between the warm subtropical and cold subpolar gyres, topographic form stress and steering, and fine-scale eddy transport and mixing [3,4]. Adding to the complexity, the exchange between the surface and interior ocean is mediated by the surface mixed layer, which itself responds to seasonal buoyancy forcing, wind stress, lateral mixing and upwelling [5], as well as isopycnal [6] and diapycnal mixing [7] and wind-driven pumping in the ocean interior. The system is complicated further by the presence of Antarctica to the south, with its massive ice-sheet freshwater reservoir and seasonal sea-ice extent (SIE). Although much progress has been made in recent years towards understanding the interplay of these factors through vastly increased numbers of high-quality observations [8] and concerted modelling efforts, the response of the system to projected anthropogenic climate forcing still has a great deal of uncertainty. One way of addressing this uncertainty is through the use of large numbers of climate models and looking for robust trends to emerge across the ensemble.

The Coupled Model Intercomparison Project phase 5 (CMIP5) is an international collaborative effort to develop such coupled climate model simulations within a coordinated experimental framework. It aims to provide a multi-model suite of projections of the Earth's climate under a number of specified anthropogenic forcing scenarios in order to assess climate response, gauge its predictability, identify feedback cycles and understand the factors that drive differences between models [9]. CMIP5 builds on the success of its predecessor, the Coupled Model Intercomparison Project phase 3 (CMIP3) [10], which made significant contributions to the Intergovernmental Panel on Climate Change's (IPCC) Fourth Assessment Report (AR4) [11]. CMIP5 improves on CMIP3 in a number of areas: significantly improved spatial resolution in both atmosphere and ocean (although the ocean remains too coarse to permit mesoscale eddies), a larger proportion of full Earth system models (ESMs) resolving biogeochemical cycles, the inclusion of atmospheric ozone in all models and more complete atmospheric chemistry, and a dramatically expanded list of available ocean, atmosphere and cryosphere variables. These improvements mean that over 100 times more data are archived (more than 3 PB) and have been made available from CMIP5 relative to CMIP3 [9].

The Southern Ocean was the focus of much attention in CMIP3 owing to its important role in the global climate system and because it is the site of one of the largest observed climate trends. The strong positive Southern Annular Mode (SAM) trend [12] has been linked to greenhouse and ozone forcing [13] and has led to a poleward shift and strengthening of the mid-latitude westerly wind jet. This, in turn, has been linked to oceanic changes in ACC frontal positions [14] and water mass property changes [15–17]. Results from CMIP3 indicate that the wind trend will be continued into the twenty-first century [18,19] and that there is an associated poleward shift of the ACC, along with general warming of the upper ocean, a freshening of intermediate layers [20] and an intensification of the upper MOC [21]. The oceanic warming is associated with a reduction in SIE [22] and consequent slowdown of Antarctic bottom water (AABW) formation close to the continent [20]. Despite these consistent trends, CMIP3 also produced a number of inconclusive results, and demonstrated a worryingly wide inter-model spread of ACC and MOC strengths and water mass characteristics and distribution, particularly for Antarctic intermediate water (AAIW). The CMIP3 models also failed to reproduce the observed expansion of SIE over the altimetric record [22] and did a generally poor job of reproducing its distribution or total extent. Russell [23] found that only one of 18 models assessed was not deficient in at least one area of Southern Ocean simulation.

The output of up to 61 CMIP5 models and hundreds of variables has been generally available for almost 2 years now and enough publications on the representation of various aspects of the Southern Ocean in CMIP5 under climate forcing have emerged to begin to form a coherent assessment of the state of the art in coupled climate modelling for this region. This review seeks to summarize the findings of these studies and draw them together to provide an overview of the representation of the present-day Southern Ocean, how it is projected to change under climate forcing scenarios and how this has changed relative to CMIP3. These studies all examine the output of the historical (HIST) scenario that aims to re-create present-day climate with observed forcing between 1850 and 2005. Most also examine the representative concentration pathway (RCP) future scenarios that specify concentration and emissions of greenhouse gases (GHGs) from 2006 to 2300, although most studies focus on end of twenty-first century changes. A full range of anthropogenic climate drivers are included in the RCP scenarios (GHGs, aerosols, chemically active gases and land use) along with a repeating 11-year solar cycle [24]. In these studies, two main future scenarios are examined: RCP4.5, an intermediate-emissions scenario, and RCP8.5, a high-emissions scenario, where 4.5/8.5 represent the approximate increases in radiative forcing (W m^−2^) induced by the year 2100 under these scenarios.

This review first examines the Southern Ocean wind stress forcing as it relates to the ocean and sea ice (§2), followed by the dynamical representation of the ACC and MOC (§3) and the Southern Ocean bulk properties and thermodynamics in §4. It concludes with an examination of the representation of sea ice and the subpolar regime (§5) and a discussion of the system as a whole, what questions remain open and approaches to future research (§6). A representative sample of the CMIP5 models most often examined in the studies reviewed here is given in table 1. Listed in table 1 are some of the key model methodologies, treatments of the ocean or atmosphere, and parametrizations pertinent to the Southern Ocean, such as ocean and atmosphere resolution, ocean vertical grid type, the resolution of the stratosphere, etc. The relative impact of these inter-model differences can be hard to disentangle owing to the number of other factors that also vary between models. For example, some authors [32,34] could find no clear ocean resolution dependence for ACC transport or core latitude, and similarly no obvious differences between full ESMs and those that did not include explicit carbon cycles. In other cases, however, model set-up was found to have a significant impact on the representation of the Southern Ocean. These are discussed more fully in the relevant sections in the following.

**Table 1.** Details of a representative selection of the models used in the studies referenced in this review. ESM indicates if the model includes a coupled carbon cycle. Hi-top indicates stratospheric resolution (model tops at or above 1 hPa). Atmospheric resolution is in degrees. Ocean grid refers to the vertical coordinate, where *Z* indicates constant depth levels and *σ* isopycnic coordinates (*σ*–*Z* are hybrids). Ocean resolution is zonal mean ocean grid longitude and latitude differences at 50°S. *κ*(m^2^ s^−1^) indicates minimum and maximum allowed thickness diffusion coefficients for the eddy parametrizations. References for eddy schemes: Griff98 [25] Griff05 [26], GM90 [27], GM95 [28], Vis97 [29], Treg97 [30] and Eden08 [31]. Details compiled from [32,33] and personal communications with respective modelling groups.

model name	ESM?	hi-top?	atm. res.	ocean grid	ocean res.	*κ*(m^2^ s^−1^)	eddy param.
BCC-CSM1-1	Y		2.8	*Z*	1.0×1.0	100–800	Griff05, Griff98
CanESM2	Y	Y	2.8	*Z*	1.41×0.93	1000	GM95
CNRM-CM5	Y	Y	1.4	*Z*	1.0×0.65	15–3000	GM90
CSIRO-Mk3.6			1.9	*Z*	1.88×0.93	50–600	Vis97, Griff98
GFDL-ESM2G	Y		2.0	*σ*	1.0×1.0	10–900	GM90
GFDL-ESM2M	Y		2.0	*Z*	1.0×1.0	100–800	Griff05, Griff98
GISS-E2-H		Y	2.0	*σ*–*Z*	1.0×1.0	?	?
GISS-E2-R		Y	2.0	*Z*	1.25×1.0	?	GM90
HadGEM2-CC	Y	Y	1.25	*Z*	1.0×1.0	150–2000	Vis97, Griff98
HadGEM2-ES	Y		1.25	*Z*	1.0×1.0	150–2000	Vis97, Griff98
INMCM4	Y		1.5	*σ*	1.0×0.47	?	?
IPSL-CM5A-LR	Y	Y	1.9	*Z*	1.98×1.30	15–3000	Treg97
IPSL-CM5A-MR	Y	Y	1.3	*Z*	1.98×1.30	15–3000	Treg97
IPSL-CM5B-MR	Y	Y	1.3	*Z*	1.98×1.30	15–3000	Treg97
MIROC5			1.4	*σ*–*Z*	1.41×0.78	300	GM95
MIROC-ESM	Y	Y	2.8	*σ*–*Z*	1.41×0.93	700	GM95
MIROC-ESM-CHEM	Y	Y	2.8	*σ*–*Z*	1.41×0.93	700	GM95
MPI-ESM-LR	Y	Y	1.9	*Z*	1.41×0.89	100	GM95
MRI-CGCM3	Y	Y	1.1	*Z*	1.0×0.5	300–1500	Vis98
NorESM1-M	Y		1.9	*σ*	1.13×0.53	?	Eden08

## 2. Southern Ocean winds

The Southern Ocean circulation is directly influenced by the momentum transfer of the strong westerly winds that exist over the ACC and by the easterly winds closer to the Antarctic continent. Both the ACC isopycnal gradient, and therefore its zonal circulation, and MOC have strong wind-forced components, although the exact coupling between wind and buoyancy effects is difficult to disentangle. Winds also directly influence mixed layer depths, vertical Ekman pumping and sea surface temperature, and therefore impact on oceanic carbon and oxygen uptake and storage. It is therefore critical that these winds are modelled accurately in order to assess the response of the Southern Ocean to predicted climate forcing.

**Figure 1.**
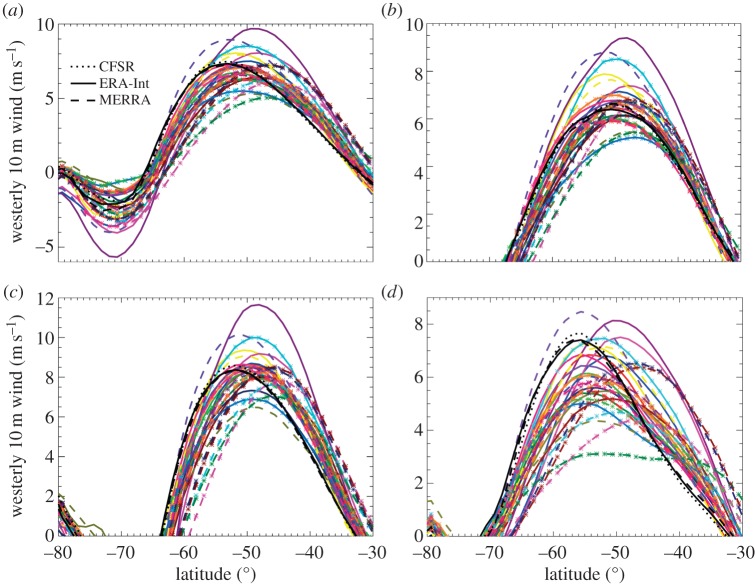
(*a*) Zonal mean annual mean 10 m westerly wind climatology for the period 1985–2004. (*b*–*d*) The same but for the Atlantic, Indian and Pacific sectors, respectively. The coloured solid and dashed lines indicate the CMIP5 models, whereas the black lines show output from the observations and reanalyses listed in (*a*). Reproduced with permission from [33].

Under the HIST scenario between 1985 and 2004, for the multi-model mean of 29 CMIP5 models, the peak strength of the westerly wind jet is biased 3.3±1.9° equatorward relative to reanalysis products, with no ensemble members peaking south of the observed position (figure 1) [33]. This bias is most pronounced in the Indian and Pacific basins, but is also large and positive (1.4°) over the Atlantic. The bias tends to be smaller in atmosphere-only runs of a subset of the same models, suggesting that coupled atmosphere–ocean feedbacks may be partially contributing to this error, but more specific causes are not yet apparent. The jet peak strengths tend also to be biased low (−0.4±1.0 m s^−1^) relative to reanalyses, although there is more inter-model variability and biases tend to be positive (0.3±1.0 m s^−1^) in the Atlantic and strongly negative (−1.4±1.0 m s^−1^) in the Pacific sectors. It is worth noting that, while there is some uncertainty in the accuracy of reanalysis datasets, the variability between three reanalysis products compared by Bracegirdle *et al.* [33] was negligible in comparison with inter-model differences, even taking into account the fact that the reanalysis-derived uncertainty is probably slightly smaller than actual observational uncertainties [35].

Under the RCP4.5 and RCP8.5 scenarios, there is a clear trend in westerly winds towards stronger and more poleward jets, with a directly associated positive trend in the SAM [36]. This proceeds in three broad phases: an increase in strength and poleward shift in the late twentieth century, a weaker change over the first half of the twenty-first century and then a clear poleward shift and strengthening winds in the latter half of the twenty-first century. The plateau at the start of the twenty-first century is probably due to the recovery of the Austral summer ozone hole counteracting carbon forcing during this period [33]. This shows that the inclusion of ozone forcing has had a measurable impact on wind stress [37], although, in terms of position and jet strength, this effect is most pronounced in weaker RCP scenarios; and under RCP8.5, the effect of ozone recovery on jet position is almost negligible relative to GHG forcing [38]. The annular component of the SAM is relatively well re-created in CMIP5, with spatial correlation coefficients between observations and an ensemble of 12 CMIP5 models generally being larger than 0.85, which also represents an improvement over CMIP3 [39]. While the zonal mean structure of the SAM is in agreement with observations, the non-annular components are less well represented. This is particularly the case for the cyclonic circulation north of West Antarctica, where the core longitude varies between models by up to 90° and causes considerable variability to climate over the Antarctic Peninsula [40].

Climate forcing of the atmospheric westerly jet results in a strengthening of over 10% and a poleward shift of over 1.5° by the end of the twenty-first century in CMIP5 [38]. There is considerable variability between models in the degree of both the strength and position change, with large inter-basin variability, although the sign is clear in all models and associated with a positive SAM trend [39]. A state bias exists for the jet response, with those models with the largest initial biases in position being associated with the largest poleward shifts (*r*≈−0.6 [33,41]), meaning that future jet shifts may be overestimated owing to initial equatorward biases in jet representation. The reason for this bias is as yet unclear, but may relate to differences in dynamical regimes across the major ocean basins [33].

Some inter-model variability may partially be explained by the difference in stratospheric resolution between models, with some having ‘high tops’ and others ‘low tops’ that do not fully resolve the stratosphere (table 1). Some correlation does exist between stronger jet position responses to climate forcing and those models that resolve the stratosphere, possibly linked to upper-troposphere tropical warming, but this correlation does not extend to jet strength [33,41] or model skill at representing the SAM [39]. Despite the improvements in atmospheric chemistry and vertical resolution of CMIP5 over CMIP3, there is little significant difference in the representation of the surface wind stress between the two model ensembles [42] and the bias in the westerly wind stress peak strength and latitude continues to be a problem in CMIP5 [33].

## 3. Southern Ocean circulation

### (a) Horizontal circulation

Improvements have been made over CMIP3 in re-creating both the mean path and strength of the ACC. Improved topographic constraints due to better-resolved bottom topography largely ensure that the mean latitude of the ACC core lies within one degree (0.06±1.27°) of the observed ACC core in CMIP5 models in the HIST scenario between 1976 and 2005 [32]. Regional variability from the observed mean position is generally relatively small, and the equatorward bias seen in the western Atlantic in CMIP3 has largely been corrected in CMIP5, although the ACC core still tends to be biased equatorwards in the eastern Pacific. In CMIP3, there was found to be a strong and statistically significant correlation between the position of the ACC and the position of the zonal wind stress maxima [20,23]. This relationship does not appear in CMIP5, where for an ensemble of 22 models there is no significant correlation between the position of the ACC and either the strength or position of the westerly wind stress maxima in the HIST scenario.

The volume transport through the Drake Passage is the focus of many observational studies (134–164 Sv [43]), but it was poorly re-created in CMIP3, with an extremely large range of values (33–337 Sv, mean 144.6±74.1 Sv, for a 19 model ensemble) [20, 23]. CMIP5 still has a significant range (90–263 Sv across 23 models [32]), but they are tending to converge (mean 155±51 Sv), with only three models greater than 200 Sv. There has been relatively little change in surface wind stress strength or position (§2), so this improvement must reflect some other model change. One such candidate may be the broad implementation of more sophisticated Gent–McWilliams [27] (GM) eddy parametrization schemes. In CMIP3, there was a strong relationship between the ACC transport and the eddy-induced thickness diffusivity (*κ*) for those GM schemes that used a fixed coefficient [44]. These simpler schemes are almost entirely replaced in CMIP5 with more complex parametrizations where the diffusion coefficient is itself a function of the density field, such as the Visbeck parametrization [29]. The *κ* value may range by up to two orders of magnitude between models (table 1) and vary in space and time. As well as improving the parametrizations themselves, a realization of the generally poor model performance in the ACC in CMIP3 also probably prompted a greater focus on tuning these parametrizations for more accurate transports. The increase in model resolution and therefore bathymetry is also likely to have contributed to the improved ACC transport in CMIP5, although no models have yet increased resolution to the extent that eddy dynamics are represented, and no resolution dependence for ACC position or strength is apparent [32].

Under the RCP4.5 and RCP8.5 forcing scenarios, the ACC change over 2000–2099 in CMIP5 is largely similar to that seen in CMIP3, with a few notable differences. In CMIP5, there is no clear relationship between the coherent wind stress changes (§2) and either the transport or mean latitude of the ACC. There are significant changes in individual models, but no coherence across the ensemble (−2.7±9.8 Sv and 0.01±0.44°, respectively, for RCP8.5 [32]). This is perhaps unsurprising, as, although non-eddy-resolving ACC models have been shown to respond strongly to increases in wind forcing in idealized experiments [45], the wind stress change under future climate forcing is significantly smaller (order 10%) than the multiple factors applied in such experiments. In CMIP3, there was also no correlation between ACC transport and wind stress [20], but the ACC position was much more variable than in CMIP5 and moved in response to the position of the westerly wind stress maxima, in some cases by up to 4° [20]. Why this does not occur in CMIP5 is presently uncertain but may be due to increased topographic pinning by better-resolved bathymetry.

So what does drive the sometimes large (−26 to 17 Sv) changes in ACC transport in some models if not changes in surface wind stress? This is presently an open question, although some clues exist. In both CMIP3 and CMIP5, there is a strong correlation between the change in ACC transport and change in the area of the ACC itself, with narrowing ACCs tending to have weaker transport [32,46]. In CMIP5, the northern boundary of the ACC is universally moved polewards by the expanding subtropical gyres, themselves forced by increases in wind stress curl. However, the degree of poleward shift is not correlated with the change in ACC transport. Instead, it is the change in subpolar gyre area that correlates (*r*=0.67 in RCP4.5) with the change in transport, with weakening ACCs being associated with equatorward expanding subpolar gyres. Those gyres that increase in size also increase in strength [47]. Although the changes in area can be significant, there is no clear pattern to the changes in the subpolar gyres, with the Ross and Weddell Gyres appearing to behave independently both within and across models [32]. The Ross Gyre tends to exhibit the largest degree of variability and qualitative influence on the ACC position, perhaps as a result of weaker topographic constraints in the Pacific sector. As in CMIP3, changes in wind stress curl are not correlated with the gyre strength [48], despite high-resolution model studies suggesting that wind stress curl is a significant factor in setting gyre strength [49]. Ekman pumping, ACC eddy interaction and surface freshwater fluxes have all been suggested as possible factors controlling the subpolar gyres [47,48], but no clear cause has yet been identified. A further complicating factor may be the presence of physically unrealistic open water convection occurring in the Weddell and Ross Gyres in CMIP5 [50] that will certainly impact the gyre transport and density structure, although the extent of this has not yet been investigated.

The change in the width of the ACC through movement of its southern boundary will not necessarily reduce baroclinic transport, however, if the mean meridional density difference remains constant. A number of other processes may therefore be contributing to the observed inter-model transport variability. Increased *κ* will reduce isopycnal tilt over mid-depths in the ACC, suggesting that, if the ACC northern and southern boundaries narrow, increased isopycnal gradients may be reduced by enhanced GM transport, depending on the eddy parametrization implemented in each model [44]. Changes in wind stress curl and associated Ekman pumping may also modify isopycnal slopes, and, owing to the stronger vertical stratification north of the ACC, changes in pumping here may be more effective in modifying ACC transport than south of the current [46]. Despite this, Ekman pumping does not appear to directly modify transport in CMIP3, but rather it is the buoyancy fluxes of heat and freshwater in the upwelling and downwelling regions north and south of the ACC that most strongly correlate with transport [51]. It is uncertain whether this relationship also holds in CMIP5.

### (b) Vertical circulation

The Southern Ocean MOC may be divided into two component cells that oppose each other to produce a residual, net, overturning [6]. The Eulerian circulation is principally wind-driven and moves water equatorward in the Ekman layer, tilting up isopycnals in the process, and returns it at depth supported by geostrophic pressure gradients below the shallowest topography. This is opposed by an eddy-driven circulation, parametrized in CMIP5 models as some form of GM transport, that fluxes thickness downgradient to reduce isopycnal tilt. The residual circulation is extremely difficult to observe, and assessing the modelled eddy circulation requires the output of either product terms (e.g. 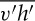) or high-temporal-resolution velocity and tracer fields. Unfortunately, these were not required variables in CMIP5 and so cannot be readily assessed. The zonal mean overturning circulation was an output variable, although with a low priority of 3, and so only a handful of groups made these data available. A study of eight models found that there is a large range of both Eulerian and eddy cell transports for both the upper and lower overturning cells, with differences of over 200% between the weakest and strongest eddy contributions in the lower cell [52]. The upper cell residual circulation is similar to observational inverse estimates (15 Sv, [53]), whereas the lower cell tends to be stronger than observed. The small sample size and large inter-model variability make accurate assessment difficult, but this residual circulation is generally consistent with water mass budgets based on larger numbers of CMIP5 models (§4*b*) [54]. The choice of *Z*-level or isopycnic vertical ocean coordinates (table 1) does not appear to make a dramatic difference to the MOC [52], but this could be a result of too small a sample of models.

Under RCP8.5, the wind-driven upper cell increases in strength by up to 20% in proportional response to the general strengthening of the wind stress maxima, while the lower cell weakens by 10–60% by the end of the twenty-first century [52]. Eddy compensation of these changes occurs in both upper and lower cells but is only partial and varies significantly between models. This results in a net strengthening of the upper cell and weakening of the lower, which is qualitatively consistent with water mass budget analysis [54]. In their analysis of four CMIP5 models, Downes & Hogg [52] find that the MOC changes are most consistently related to wind stress change, but the small sample size makes the response to changed surface buoyancy fluxes uncertain. These results are broadly consistent with the examination of the Eulerian MOC in CMIP3 models where the Deacon cell spins up and its maximum shifts uniformly in response to stronger and more poleward westerly winds, whereas the bottom water cell is reduced in strength by around 2 Sv [20]. It is difficult to assess the impact that improved eddy parametrizations has had on the MOC as eddy circulation statistics were not available in CMIP3.

## 4. Water mass properties and change

### (a) Water mass bulk properties

**Figure 2.**
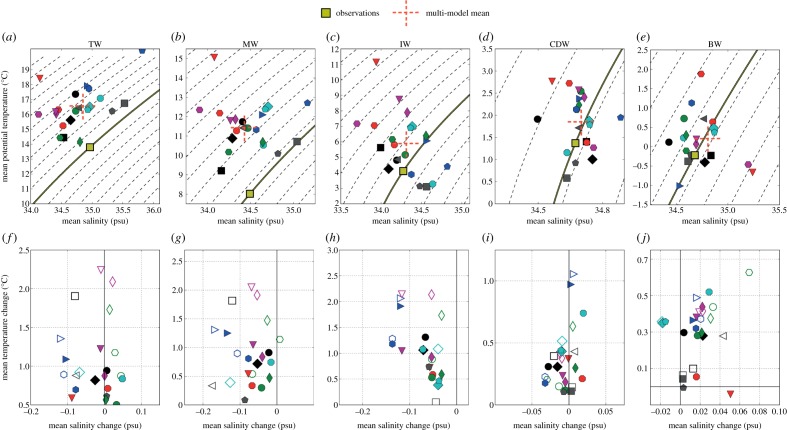
Southern Ocean temperature–salinity characteristics and future changes. (*a*–*e*) The mean temperature–salinity of each water mass for the period 1970–2000 with superimposed 0.2 kg m^−3^ density contours. (*f*–*j*) The mean end of twenty-first century change in the temperature and salinity characteristic (2070–2100 minus 1970–2000). The filled symbols represent changes associated with scenario RCP4.5, and the unfilled symbols represent changes associated with scenario RCP8.5. Each column is associated with one water mass: from left to right, subtropical water (TW), mode water (MW), intermediate water (IW), circumpolar deep water (CDW) and bottom water (BW). Adapted from [54].

Owing to the numerous processes influencing water mass formation, mixing and export, the accurate representation of even the present state of the Southern Ocean has been challenging in coupled climate models, and in past efforts, there have been significant differences between models and observational estimates [21,51]. Sallée *et al.* [54] use dynamically based water mass definitions rather than fixed density classes to uniformly assess the water masses across 21 CMIP5 models in the HIST scenario. They find that similar variability is present in CMIP5 (figure 2).

In common with CMIP3 [55], the sub-Antarctic mode water (SAMW) and AAIW are generally poorly represented. The potential vorticity minimum is found to be too light and in different locations to observations. The winter-time mixed layer is generally biased too far north and too deep, particularly in the western Indian and Pacific basins. This results in subtropical mode waters rather than SAMW, which subsequently penetrates less deeply and at lighter water mass classes, as also occurred in CMIP3 [56]. This light bias is itself a result of large inputs of heat and freshwater (multi-model mean salinity of 34.5 Sp and temperature of 11.4°C relative to observations of 34.6 Sp and 9.2°C, respectively) in the SAMW formation regions [34]. AAIW forms as the densest class of SAMW in a relatively small region to the west of and in the Drake Passage, and the characteristics of AAIW in CMIP5 models are closely linked to the depth and properties of the mixed layers of this region [34]. The AAIW freshwater and low-potential-vorticity tongue is generally well represented and closer to observations than the SAMW, although still tending to be biased too light by 0.2 *kg* m^−3^. The saline bias in AAIW observed in many CMIP3 models [23] is significantly reduced in CMIP5, possibly reflecting the more accurate representation of outcrop area [54] and winter-time mixed layer depths in the south-east Pacific where AAIW is primarily formed [34].

The circumpolar deep water (CDW) has smaller biases relative to observations, although it still tends to be too warm (1.9°C relative to observational estimates of 1.3°C) and light. This probably reflects the distant formation of the CDW and the relatively weaker influence of biases in air–sea fluxes when compared with the SAMW/AAIW above and AABW below. The AABW exhibits the most dramatic variability across models of all the water masses. Its volume ranges from 3×10^16^ to 14×10^16^ m^3^, with the multi-model mean being approximately 20% larger than the observational estimate. The water mass characteristics are similarly poorly simulated, with fresh or saline biases of over 0.9 Sp from the observed value in some models, but overall there tends to be a warm bias of 0.4°C and a salty bias of 0.6 Sp [54]. These dramatic differences reflect the well-known problem of accurately capturing the complex and small-scale interactions that drive the formation of AABW (§5*b*).

Under RCP4.5, there is a very consistent warming of the water column by the end of the century of between 1 and 1.3°C, concentrated largely in the subtropical, mode and intermediate waters, with RCP8.5 warming being around 30–60% stronger [54]. Salinity changes are less vertically coherent, with consistently strong freshening of the SAMW and AAIW by 0.03 and 0.06 Sp respectively (double this for RCP8.5), driven by increases in freshwater fluxes into their formation regions [34]. CDW changes by the end of the twenty-first century tend to be less dramatic, probably due to the relatively long lag in propagating changes from the North Atlantic to the Southern Ocean. By contrast, the AABW experiences a significant increase in salinity of around 0.02 Sp and warms by over 0.3°C. Although the warming tends to agree with observations [57], the freshening is in opposition to the observed trend [58] and may be a result of the lack of ice-shelf interaction in CMIP5 models or possibly enhanced sea-ice formation in winter owing to reduced summer SIEs driving enhanced brine rejection [54].

These water mass changes, which are of relatively consistent sign across CMIP5 models, are in general agreement with the changes observed in CMIP3 [20,21]. The only exception appears to be the CDW, which exhibits a weak salinification in CMIP3, whereas, in CMIP5, there is a weak (around 0.01 Sp in most models) freshening. This may be due to the water mass definitions used [54], which may conflate some CDW with AABW where there is a clear salinification trend. The persistent warming of the water column in CMIP5 comes in all models despite a varied ACC positional response. This puts a stronger emphasis on surface warming driving the deep trend than in CMIP3, where it was argued that the more coherent poleward shift of water masses could account for much of the warming [20]. The heat uptake by the Southern Ocean under climate forcing represents up to 40% of the total global value in CMIP5 [59], with global heat uptake peaking above 700 m in the SAMW/AAIW formation regions, and on the southern flank of the ACC below 2000 m. There are no dramatic differences between CMIP3 and CMIP5 in terms of heat uptake efficiency, but there is a clear link between those models with weaker stratification and enhanced heat uptake. This in turn may be linked to the eddy diffusivity coefficient, whereby weaker *κ* lead to steeper isopycnals, weaker vertical density gradients and hence enhanced heat uptake [59].

### (b) Water mass formation and transformation

**Figure 3.**
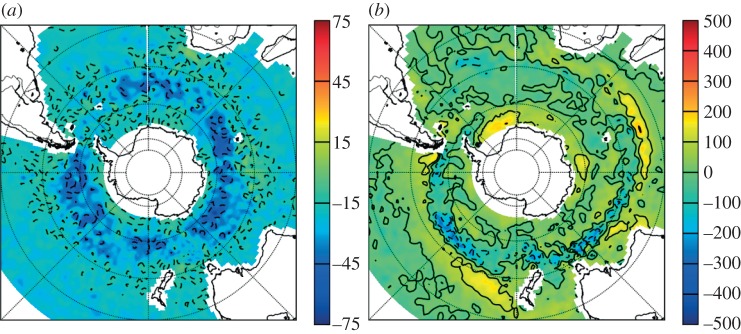
(*a*) Summer and (*b*) winter multi-model mean MLD bias relative to Argo observations (m). Negative values indicate a shallow model bias. Adapted from [34].

The water mass properties described above are controlled by surface buoyancy fluxes and mixing in the ocean interior and seasonal surface mixed layer. The mixed layer, in particular, is fundamental to the formation and SAMW and AAIW and subsequent entrainment of surface properties into the deep ocean. In CMIP5, the Austral summer mixed layer depth (MLD) tends to be around 50 m, except in the band between 50 and 60°S where depths reach 60–90 m [34]. Compared with Argo observations, this tends to be biased shallow by around 50–70 m (figure 3). In Austral winter, the deeper central band narrows and shifts equatorwards onto the northern edge of the ACC, deepening to as much as 400–700 m. The regions of deep convection extend much further into the subtropics than do observations and contribute to the over-warm SAMW and AAIW and the subduction of subtropical mode water identified in the previous section. Despite the wider extent of deep mixed layers, the multi-model mean in winter is still 100–200 m shallower, on average, than observations. Although qualitatively there appears to be an improvement in MLD spatial distribution relative to CMIP3 [20], there remains significant spatial variability of MLD between models, particularly of the winter maxima. The spatial accuracy of the band of MLD maxima associated with SAMW and AAIW formation varies considerably between models, with a range of spatial correlations with observations ranging from 0.2 to 0.8 [34]. Errors are notable in the Indian Ocean sector where the MLD maximum is biased too far equatorward in many models, and similarly in the Pacific where deep mixed layers do not extend far enough westward. In some models, these northward biases appear to be correlated with the equatorward bias of the wind stress maxima, but, in others, large MLD position biases exist where the wind stress maximum is better represented.

Under climate forcing, almost all CMIP5 models simulate a freshening and warming of the maximum MLD that is stronger under RCP8.5 than RCP4.5 [34]. This leads to a shoaling of the MLD maximum by up to 30 m (mean 20 m) and 100 m (mean 40 m) for RCP4.5 and RCP8.5, respectively. This appears to be driven by a reduction in the winter-time buoyancy loss, particularly in the SAMW/AAIW formation regions (*r*=−0.63). This shoaling was also observed in CMIP3 [20,55]. Interestingly, the response of the MLD to surface warming and freshening exhibits a strong state dependence, with those models with the shallowest MLD also displaying the smallest shoaling, whereas deeper initial conditions result in larger reductions. This is particularly the case in the eastern Pacific, where the correlation between initial state and the magnitude of change is *r*=0.82 [34]. This state bias may mean that future shoaling is underestimated owing to the shallow bias in HIST MLD.

So why is the MLD biased shallow in the Southern Ocean, and what are the implications for the ventilation of the ocean interior? Unlike observations where air–sea buoyancy fluxes along with Ekman contributions are found to be critical in setting the mixed layer properties [60], there is only a weak anticorrelation between surface buoyancy fluxes and MLD in CMIP5, and none at all when Ekman fluxes are included [34]. Instead, in regions of deep winter MLD where SAMW and AAIW form, the stratification at the base of the winter mixed layer is more than twice that of observations. In the models with the most biased MLD, this stratification tends to arise due to a positive (fresh and stable) bias in the haline component. The strength of the haline bias explains much of the inter-model MLD variance (*r*=0.5), whereas the thermal stratification plays a negligible role. Despite their weak relationship with the MLD itself, the combined air–sea heat and freshwater fluxes are strongly (*r*=0.9) related to the stratification at the base of the mixed layer. In general, the freshwater buoyancy component (44 W m^−2^) dominates the thermal component (−31 W m^−2^) in the SAMW/AAIW formation regions. The poorly known observational value of surface heat and freshwater fluxes means that it is presently difficult to assess the relative accuracy of these model values. Strong increases in both surface heat and freshwater fluxes at high latitudes are predicted under future climate forcing [61], but, despite the strong relationship between surface buoyancy fluxes and stratification at the base of the mixed layer in the HIST scenario, there does not appear to be a coherent or large change in stratification at the base of the mixed layer under future climate forcing, resulting in the dominance of surface buoyancy fluxes in controlling MLD properties under future climate forcing. Another possible cause of the shallow bias may be due to underestimating the high-frequency wind variability [62], although this has not yet been investigated in depth in CMIP5.

The properties of the SAMW and AAIW are tightly coupled with the characteristics of the mixed layers in which they form. In the regions of SAMW formation, the MLD is correlated with the total volume of subducted water masses, and the predicted shoaling of MLD in response to future climate forcing drives a corresponding (*r*=0.81) reduction in volume of these water masses, as also seen in CMIP3 [20,52]. The change in MLD and reduction in water mass volume is also linked to the total outcropping area of water masses [54]. The outcropping regions of SAMW and AAIW are reduced by around 10%, with these losses being replaced by increased outcropping regions of subtropical water and CDW. The poleward shift of the subtropical gyres and water outcrop region is consistent with this change [32].

**Figure 4.**
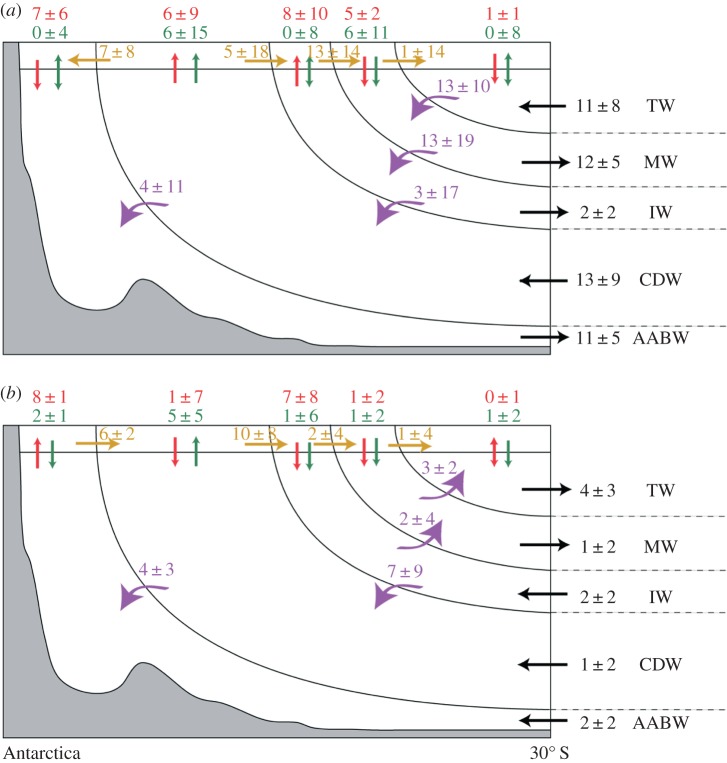
Water mass formation, transformation and export out of the Southern Ocean. (*a*) Schematic showing the multi-model mean formation, transformation and transport of water masses across 30°S: formation from heat flux (red) and freshwater flux (green); transformation from surface buoyancy flux (yellow) and interior processes (purple); export across the 30°S boundary (black). (*b*) As for (*a*) but for changes under the RCP8.5 scenario (2070–2100 minus 1970–2000). All numbers are in Sv. Adapted from [54].

The volumetric exchange between water masses in the HIST scenario of CMIP5 was investigated by applying a Walin [63] water mass budget to the surface buoyancy flux, water mass volumes and transport across 30°S (figure 4) [54]. Upwelled CDW/AAIW is transformed to SAMW and around 7 Sv of CDW to AABW through surface buoyancy fluxes. Interestingly, no net AAIW is formed directly on the surface due to buoyancy fluxes, and instead an upwelling of around 6 Sv occurs in this class and around 13 Sv is converted to modified CDW densities, where a total of around 11 Sv is subsequently subducted and exported northwards. Interior mixing tends to transform this subducted SAMW back into AAIW classes. The net formation of AAIW from SAMW via ocean interior processes is consistent with CMIP3 [51] and observational studies [5], but this bulk view hides much regional variability, notably AAIW forming through surface forcing near the Drake Passage.

There is very large inter-model variability in the numbers associated with both formation and destruction of water masses on the surface and interior processes, with the variability often significantly larger than the multi-model mean (figure 4). The largest uncertainties tend to be associated with surface freshwater fluxes, but if extreme model outliers are removed, then there is firm agreement in the dominant contribution of freshwater fluxes to the upwelling of CDW and subduction of SAMW across models. Under the RCP4.5/8.5 scenarios, the upwelling of CDW increases, largely driven by enhanced (7±9 *Sv*) interior transformation of AAIW to CDW. This extra AAIW is formed through enhanced surface fluxes of CDW to AAIW classes driving greater AAIW subduction. The enhanced subduction balances the acceleration of the upper cell of the MOC determined from the residual circulation by Downes *et al*. [52], although no significant change in transport occurs across 30°S. This implies that the enhanced circulation is confined to the Southern Ocean through enhanced interior mixing, though where and by what process are still unclear. The formation of AABW due to surface buoyancy fluxes and its export across 30°S is reduced under both RCP4.5 and RCP8.5, principally due to enhanced surface warming.

## 5. Subpolar representation

South of the ACC the subpolar regime consists of the Antarctic shelf seas, as well as the Ross, Weddell and Australian–Antarctic Gyres. The huge seasonal cycle of sea-ice cover in these regions has a dramatic impact on the thermal and hydrological cycles of the Southern Ocean and on the formation and export of AABW. The fine balance and small scale of interactions between atmosphere, ocean, sea ice, ice shelves and ice sheets (not included explicitly in CMIP3 or CMIP5) means that this region is extremely challenging to model accurately.

### (a) Sea ice

**Figure 5.**
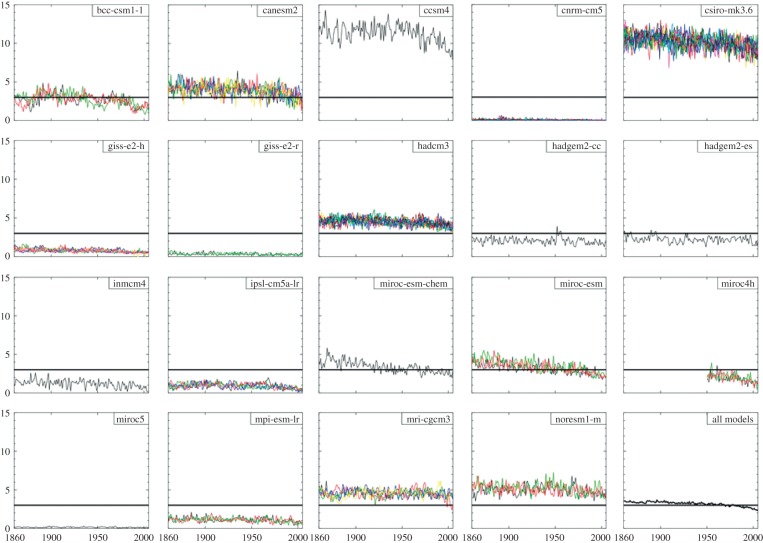
The February SIE from 1860 to 2005 as simulated by various CMIP5 models. All ensemble members are plotted. The vertical scale is 10^6^ km^2^. The horizontal line indicates the mean (1979–2005) satellite-observed SIE of 3×10^6^ km^2^ for the month of February. Adapted from [66].

In contrast to Arctic SIE, which has consistently decreased in both observations and CMIP3/5 [64], Antarctic SIE has shown a statistically significant increase over the past 30 years for reasons that are still uncertain [65]. An assessment of the SIE in 19 CMIP5 models in the HIST scenario over 1860–2005, with a focus on the period 1979–2005, finds that the majority of models do a poor job in re-creating both the seasonal cycle and the maximum extent of observations [66]. The multi-model mean SIE between 1979 and 2005 is biased below that of the observations by around 0.3×10^6^ km^2^ at the February minimum and by as much as 2.4×10^6^ km^2^ in July. The multi-model mean, which is moderately accurate [67], hides a great deal of variability between models, with some having zero SIE during the February minimum and others having three times the observed value of around 3×10^6^ km^2^. Much of this variability comes from the modelled initial state in 1979, and figure 5 shows the dramatic variability between modelled February SIE from 1860 to 2005, with differences of over 12×10^6^ km^2^ between the smallest and largest SIE. In the multi-model mean, there is a pronounced negative trend in SIE beginning after the first 50 years, which is continued into the 1979–2005 period. All bar one model have a decrease in annual mean SIE, with a multi-model mean trend of 0.33×10^6^ km^2^ decade^−1^ (−3.2%) and a peak loss of 0.4×10^6^ km^2^ decade^−1^ in September, contrasting with an observed increase of 0.29×10^6^ km^2^ decade^−1^ [66]. Examination of the spatial biases in SIE in the CMIP5 models is still preliminary, but early results reveal varied patterns of spatial variability across models, and a general failure to reproduce the largest spatial mode of change: reduction in the Amundsen–Bellingshausen Sea and expansion near the Ross Gyre [66]. This is due at least partially to the failure of CMIP5 models to consistently re-create the Amundsen–Bellingshausen Sea low-pressure system and its response to changes in the SAM. Hosking *et al.* [40] find that this bias (which varies between models and within models across seasons) also leads to correspondingly poor re-creation of surface air temperatures and errors in Antarctic climate, particularly in West Antarctica and the Antarctic Peninsula. They note, however, that the errors in the low-pressure system are at a minimum when SIE errors are at a maximum in February and warn against attributing too much of the SIE errors to winds. The trend towards SIE reduction continues into the future warming scenarios, and all but one model (in an ensemble of seven) have decreasing sea-ice volume over the twenty-first century in the RCP4.5 scenario [68].

Attributing these SIE biases and trends is difficult owing to the large number of processes that influence them. Examination of long-term unforced CMIP5 control runs shows that significant drifts do occur in these models, but that these do not have significant impact on the forced trends. Instead, the bias in absolute SIE at the start of forced integrations has a much larger impact on total SIE, and this appears to be established very quickly after the start of control runs. What causes this initial offset is unknown, although there is a correlation of around *r*=0.4 between the temperature of the deep CDW well removed from the sea-ice zone (below 2000 dbar and north of 50°S) and the mean HIST SIE, with warmer models having less sea ice. Other studies also observe this link between ocean temperatures and SIE [66], and there is a positive relationship between increased heat content in the mixed layer and reductions in sea-ice thickness under RCP4.5 forcing [68]. Ocean temperatures, and particularly the positive biases present in CDW, are therefore likely to have a significant impact on SIE representation, but the relatively weak correlation indicates that other factors are at work. These are not presently well understood, although progress is being made on the relative influence of dynamic versus thermodynamic effects [69,70].

It is possible that the observed positive SIE trend may be due to intrinsic natural variability that masks a decrease due to climate forcing. Simply based on the number of CMIP5 ensembles that increase in SIE rather than decrease in the HIST run, there is only around a one in 10 chance that the observed trend is a result of intrinsic variability aliasing the climatic trend. However, given the inaccuracy with which the models reproduce most aspects of the SIE, it seems unlikely that the CMIP5 ensemble intrinsic variability is representative of the real world and so may not be an effective way of establishing the nature of the observed trend [70,71]. Overall, the CMIP5 SIE results do not show an improvement over CMIP3 [70] and many of the individual model biases of CMIP3 are repeated in CMIP5 [66]. This is despite the improvements in ozone forcing and atmospheric chemistry in CMIP5, implying that other factors are at play in driving the observed gain of sea ice. The absence of dynamic ice-sheet melt in the HIST run does not appear to be a significant factor in the poor HIST SIE representation [72], although it may significantly impact future forcing scenarios.

### (b) Antarctic bottom water formation and subpolar circulation

As noted in §4*a*, the HIST AABW representation over the whole Southern Ocean varies significantly between models in terms of volume, temperature and salinity. This variance is due to the difficulty in accurately representing the complex chain of interactions between water masses, sea ice, winds, bathymetry and ice shelves/sheets that combine to drive AABW formation. Simplistically, AABW formation takes place over the continental shelves during Austral winter in relatively small regions of intense sea-ice formation known as polynya. Dense shelf water formed in this way either spreads over the shelves, as in the Ross and Weddell Seas, or fills bathymetric depressions, as in East Antarctica, before cascading over the shelf break and reaching abyssal depths. Ten of 15 CMIP5 models successfully form dense shelf water in Austral winter but none of these models produced AABW from shelf sources [50]. This is probably due to spurious horizontal mixing as dense water moves down the slope, resulting in the shelf water mixing with intermediate-depth water masses. Interestingly, however, the four isopycnal coordinate models tested did not perform significantly better at forming AABW than *Z*-level models, implying that the problem is likely to be a result of more than one model deficiency. Around half the models examined still have relatively realistic AABW properties, but these were produced through physically unrealistic open-ocean deep convection north of the shelf break, often to full depth. This occurs largely in the Weddell and Ross Gyres, and is a process that is very rarely observed in the Southern Ocean. The formation of these deep convection cells appears to linked to over-vigorous sea-ice seasonal cycles where summer SIE is too low, resulting in stronger than observed winter ice growth, brine rejection and hence overturning [50]. Those models with larger summer SIE had weaker deep convection, whereas those with extensive summer sea ice have none.

This physically unrealistic deep convection in the Weddell and Ross Gyres may be one possible cause of the poor relationships between wind stress curl and subpolar gyre strength reported in both CMIP3 [48] and CMIP5 [47]. It may also influence the broader structure of the gyres, and the dramatic differences in size and strength of open-ocean convection between models may go some way to explaining the large variance in strength and structure of the subpolar gyres between models. For example, in some models, a ‘supergyre’ forms, consisting of the Weddell and Australian–Antarctic Gyres, or, in extreme cases, all three gyres. The variability between models of the HIST gyre states is reduced in CMIP5 relative to CMIP3, but the inter-model standard deviations are still over 50% of the multi-model mean [47]. As noted in §3*a*, there is a strong relationship between the change in overall size of the subpolar gyres and the change in ACC transport under climate forcing, but it is unclear which variable is driving the other, or if both are responding to an outside force. Certainly, the presence of deep open-ocean convection in some models will also result in changes to the isopycnal structure- and eddy-induced transport, as well as enhancing the vertical transport of surface properties through reduced stratification. Although the role of eddies, model viscosity, bottom form stress and the presence of free circumpolar wind forced modes have all been suggested as possible contributors, there is no firm consensus on what is driving the inter-model variability of subpolar hydrography [47].

## 6. Summary and conclusions

By integrating a number of studies of the CMIP5 ensemble in the Southern Ocean together, a picture emerges of it as a ‘better CMIP3’ [73] rather than as a dramatic step forward over its predecessor. Despite the larger ensemble of models, with typically better ocean resolutions, parametrization schemes, bathymetry and a much larger proportion of full ESMs, by and large the same initial biases and coherent changes under climate forcing occur as in CMIP3. Inter-model variability in both historical and future states does tend to be smaller than in CMIP3 for many metrics, and in the face of greater model complexity, this is definitely a positive step forward. There is still significant inter-model variability in the HIST state for many important metrics (e.g. ACC transport and SIE), but the multi-model mean does tend to be approaching the observed values, emphasizing the utility of larger model ensembles. However, most of the major problems in representing the historical state and disagreements in future change seen in CMIP3 also remain.

**Figure 6.**
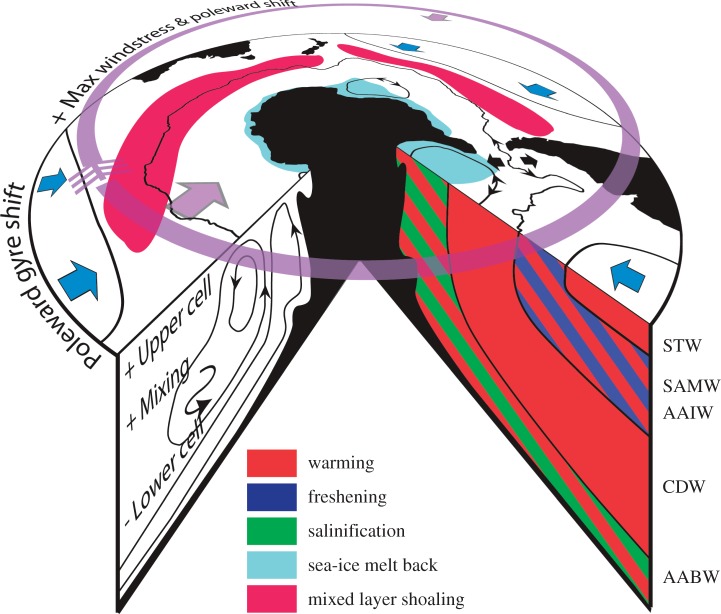
Schematic showing the impact of climate forcing in CMIP5 models in the Southern Ocean by the end of the twenty-first century. See text for more details. Arrows in both directions for the ACC and subpolar gyres indicate significant changes in transport of both signs within the model ensemble.

The most coherent features of the CMIP5 ensemble trends under the RCP4.5/8.5 forcing scenarios in the twenty-first century are summarized in figure 6. There tends not to be profound differences between the RCP4.5/8.5 scenarios, with RCP8.5 usually simply producing larger changes than RCP4.5. The HIST westerly surface wind stress over the ACC has its maximum biased equatorwards and is too weak in general. Under future forcing, all models show a poleward shift in the westerly wind stress maxima and a general increase in westerly strength, characteristic of a more positive SAM. These changes are mitigated to some extent in the early twenty-first century in weaker climate forcing scenarios by ozone recovery. The ACC itself is better represented than in CMIP3, particularly its circumpolar path, which does not appear to be strongly influenced by wind stress in CMIP5, either in its historical state or under future wind shifts. Although improved relative to CMIP3, the ACC transport still has a very large variance across models, and, under future forcing, there are significant changes in its strength, but no consistency between models. This appears to be linked to changes in the subpolar gyre area, with expanded gyres leading to weaker ACCs, but there is as yet no clear dynamic understanding of this correlation, if one component forces the other or if both are responding to external forcing. The subpolar gyres themselves have dramatically different representations across models in circulation strength, area, vertical structure and response to future forcing. By contrast, the subtropical gyres have a coherent poleward shift in response to climate forcing. Although MOC diagnostics are limited, and there is large inter-model variance, the upper cell generally increases in strength owing to enhanced wind stress and weak eddy compensation, whereas the lower cell exhibits reduced overturning. This is consistent with enhanced CDW upwelling and AAIW formation and reduced AABW formation in water mass budgets. Increased interior mixing from AAIW to CDW confines the strengthening of the upper cell to the Southern Ocean, so the impact on carbon storage may be limited.

The major water masses are generally poorly represented, with a broad warm bias throughout the water column, and SAMW/AAIW also tends to be too fresh. The mode water bias arises partially due to the mixed layer where they are formed being biased too shallow, too far equatorward and too warm. Overstrong freshwater air–sea fluxes appear to drive the shallow MLD bias via overstratification, although this is hard to verify as buoyancy flux observations are so sparse. Under future forcing, these biases are enhanced and the water column warms, mode waters freshen and the MLD shoals in almost all models, as in CMIP3. Also consistent with CMIP3 is the reduction in AABW formation and its increase in salinity. The large variability in water mass temperature may account in part for the dramatic inter-model variability in SIE, seasonal change and regional variability, although many other factors are also at play. SIE tends to be biased low in the HIST scenario, and the observed increase in recent decades is not replicated. There is no significant improvement of SIE in CMIP5 from CMIP3 and, as in CMIP3, future forcing almost universally reduces SIE.

This review highlights a number of areas where significant disagreements exist between models in both the HIST state and the modelled responses to future climate forcing. These notably include the ACC transport, surface buoyancy flux-driven water mass formation of SAMW, AAIW and AABW, SIE and subpolar gyre dynamics. These problems have many contributing and inter-related factors, but two principal problems stand out: first, the variability of both HIST and future surface heat and particularly freshwater fluxes over the Southern Ocean; and second, the difficulty of accurately representing numerous processes occurring in the subpolar zone.

The consistent changes in wind in both CMIP3 and CMIP5 but varied ACC, subpolar gyre and, to a lesser extent, MOC responses make it clear that changes in the meridional density structure are dominant over changes in the zonal wind stress in setting the ACC transport over centennial time scales. This is at least true for the relatively smaller changes in wind stress seen in the CMIP models. The fact that these models do not resolve eddies, and so the expected eddy saturation mechanism opposing wind-driven acceleration is largely absent, makes this lack of response to increased wind stress even more significant. Surface buoyancy fluxes also impact the properties of SAMW, AAIW, AABW and particularly SIE both directly in their formation regions and indirectly through setting the background stratification and impacting the mixed layer properties. This is not an easy problem to address, largely owing to the poorly observed nature of surface buoyancy fluxes in general and particularly in the Southern Ocean. Better observational and reanalysis estimates of these fluxes are essential to tuning model fluxes and hopefully providing a more consistent water mass structure in these coupled climate models, particularly in the Southern Ocean, where sloping isopycnals result in much of the water column intersecting with the ventilated layer.

The subpolar regime is the product of many different coupled processes and factors including: sea-ice formation, extent and seasonality, surface buoyancy fluxes, meridional eddy transport, surface wind stress, background stratification, mixed layer depth and seasonality, as well as topographic steering and blocking. The relative importance of each of these processes varies regionally and is not always well established observationally, and presently it is difficult to know how efforts at improving model fidelity in one aspect will influence the wider region. That said, the presence of unphysical deep open-ocean convection in many models is a worrying sign that regional dynamics needs considerable attention. Unfortunately, those models with deep convection often also achieve the most accurate AABW representation, although this probably comes at the cost of inaccurate SIE and subpolar gyre dynamics. A parametrized approach to AABW formation, or perhaps higher regional resolutions in formation areas, may be a solution to this problem. This may also go some way to improving the variability of vertical stratification south of the ACC, which presently contributes to dramatically different rates of deep warming and consequent modification of meridional density gradients, ACC transport and presumably the MOC. This impact on the Southern Ocean density gradients and MOC is also likely to have a significant impact on the subduction/outgassing of CO_2_ and other biogeochemical tracers. The potential for climate feedbacks due to changes in the MOC in ESMs is likely to be the subject of much attention in CMIP5.

The various eddy transport schemes used in CMIP5 are also likely to strongly control the ACC transport, MOC and influence meridional heat and freshwater transport. Unfortunately, despite the recommendations of earlier CMIP3 studies [46,44], neither eddy property transports nor *κ* values were made widely available in CMIP5 models, and so presently it is very difficult to examine the known sensitivity of the ACC circulation against varying *κ* using this dataset. This problem also extends to the analysis of the MOC, where eddies play a vital role in setting the residual circulation. Eddy metrics will become increasingly important in future iterations of CMIP models as they steadily increase in resolution and more models approach eddy-permitting (approx. 1/4 degree) resolutions. At this point, comprehensive eddy and *κ* statistics will be needed to determine the relative impact of resolution and parametrization schemes, as well as understanding the ACC and MOC responses to changes in climate forcing. It is therefore vital that future iterations of CMIP make both eddy statistics and the spatial pattern of parametrization schemes widely available. Bottom pressure torque has also been identified as a potentially important factor for assessing the ACC transport and gyre circulation and so should also be included in future model output [47].

Ultimately, all of the above factors are intricately linked in the Southern Ocean and a concerted effort, particularly south of the ACC, is required to disentangle the complex processes and feedbacks that control this system, which ultimately impacts SIE, the ACC and MOC and, through these, the wider climate. As models increase in complexity and resolution into the future, the addition of eddy-permitting models, dynamic ice sheets and ice shelves and more complex biogeochemistry to this picture will complicate things further. These additions increase the potential for inter-model variability in the Southern Ocean to impact climate and sea levels in highly nonlinear ways on centennial time scales, increasing overall uncertainty in global climate response to anthropogenic forcing. This highlights the need for wider and more uniformly formatted metadata availability, as well as model set-up parameters (e.g. eddy bolus diffusion coefficients) to be output as well as physical variables in order to facilitate meta-analysis of model set-up choices and their impact on the representation of the climate system.
